# A Great Social Experiment

**Published:** 1921-02-19

**Authors:** 


**THE PUBLIC WELL-BEING**—(*continued*).

### A Great Social Experiment.

### THE MEANING OF CONTINUATION SCHOOLS.

A SOCIAL experiment which will at least do its share towards altering all our problems has just been launched in London. Within the past few weeks over ten thousand scholars have been enrolled and organised in twenty-three new schools. The London youth is fortunate, though the taxpayer may not think that the good fortune extends to himself, for in other towns the continuation schools were countermanded just as they were ready to begin. In some cities—Birmingham, for instance —teachers had been appointed, premises arranged, and all was ready, when the demand for economy brought everything to a halt by suspending the Education Act of 1918, which Mr. H. A. L. Fisher left his post as head of Sheffield University to draw up and see through Parliament. London's preparations were too far ahead, and so its scheme has gone forward. Among the provisions of the Education Act was one, long desired and discussed, and of a value obvious especially to the medical profession, to keep a hand on the child at the most dangerous moment of his life, when a physical and social transformation came over him both at once. Those who have experience of working boys know how serious and how tragically overwhelming in many cases that double transformation is. The boy who is at one moment a happy child, absorbed in play, in singing, drawing, and all the pleasures of school and games, twelve months later may be merely a little copy of all the worst faults of the men among whom he has been thrown in factory and workshop, who have thoughtlessly taken no care to moderate either their language or behaviour in the presence of a child as impressionable as wax, and with only one desire—to "be a man."

#### THE CHILD'S "LIFE-LINE."

The scheme under the Act was that each child as he reached the age of fourteen, instead of being flung into industry without even a life-line to attach him to the land in which he had so long lived, should attend school each week for two periods of four hours each, either on the same or different days. The beginning was, naturally, gradual, only children who left school at Christmas being admitted, but with the end of each term in future the numbers will be added to until the present scholars have finished their two years, when the numbers leaving will balance those coming in. Already over 90 per cent. of the children who should go to these schools are in their places. Even in normal times a large number of children are slow to find work, but openings are now exceedingly scarce, and the children welcome the school as a relief from the wearisome liberty of the streets. One of the greatest difficulties of continued education has been that by the time the children were available for it they had lost the taste for schooling and found it hard to recover. These children have been transferred from the day school to the continuation school in the most natural way, and with careful guidance they should have no experience of a period when they would lose all taste for the things of the mind. We may look forward to the time when we shall have a really educated nation. That is, a nation of men and women who shall continue all through their lives to take pleasure in books, to appreciate art, and to have the ability (almost a rarity among our working classes now) of expressing themselves clearly and in a reasoned way on the subjects which interest them.

#### WHAT THE BUSINESS MAN WANTS.

The new schools are not trade schools. In some cases they have been opened by firms for their own employees. In London Harrods have such a school. In Lancashire the firm of Tootal, Broadhurst, manufacturers of cotton goods, have a model school of the highest efficiency. But even in these schools the children are not taught trade methods. The best business men realise that a child with a well-trained mind and a developed personality is more valuable to them than a child with well-trained fingers or the knack of hitting nails. Practical work is not neglected. Indeed, two of the eight hours are given over to it. But it is work which will make

**THE PUBLIC WELL-BEING**—(*continued*).

the man a good husband, and the girl a good wife, as well as a good carpenter or sempstress. Two hours are given to calculations and drawing—a subject which few children can resist. Two more hours are given to reading or history or geography, on wider and more practical lines than the child has known before; and, finally, two hours are largely devoted to physical exercise. Advocates of conscription in the interests of national development will see in this the realisation of part of their hopes. Children of fourteen just starting out to work are often submitted to strains or conditions which in a short time undo all the work of inspection and care undertaken by medical officer and nurse and teacher through many years. Usually the danger is discovered years too late, when the child is grown up and applying for sanatorium treatment or admission to hospital. But physical exercise in the continuation school will correct many of the defects due to these bad circumstances, and the medical officers will be able to protect the child from many adverse influences, and do much to bring him safely through a dangerous period. In other ways the medical profession will gain. A more intelligent population, accustomed to read and understand, will be far more amenable to treatment and far more appreciative of scientific methods. That is looking a long way ahead, but reforms like this only bear their best fruit after long growth. But in this case benefits will be immediate, and will increase with every passing year.

